# Lipid dynamics shape thermogenic adaptation in brown fat during fasting

**DOI:** 10.1016/j.jlr.2026.101147

**Published:** 2026-09-15

**Authors:** Wentong Jia

**Affiliations:** Department of Pathology and Immunology, Washington University School of Medicine, St Louis, MO, USA

Brown adipose tissue (BAT) is a specialized metabolic organ that plays an essential role in regulating systemic energy, lipid, and metabolic homeostasis (1). Unlike white adipose tissue, which primarily stores excess energy as triacylglycerol (TAG), BAT is enriched in mitochondria and has a remarkable capacity to take up and oxidize FAs to fuel thermogenesis (2). These functions become particularly important during fasting, when nutrient availability is substantially reduced. As fasting triggers lipolysis in white adipose tissue, stored TAG is broken down to release FAs and glycerol into the circulation, providing energy substrates for peripheral tissues (3). In response to this increased availability of circulating FAs, BAT shifts its substrate preference from glucose toward FAs (4). Once taken up by BAT, these FAs face two different metabolic fates: they can be reesterified into TAG and stored in lipid droplets, or delivered to mitochondria for β-oxidation. How BAT balances these competing fates to benefit systemic energy homeostasis during fasting, and what determines this metabolic decision, has remained poorly understood. The recent study by Song *et al.* (5) now provides an unexpected answer to this fundamental question.

Using a time-course fasting paradigm in wild-type mice, Song *et al*. found that BAT does not simply increase FA oxidation in response to the surge of circulating lipids. Instead, fasting progressively reprograms BAT toward lipid esterification and storage. BAT initially exhibited a modest lipolysis during the first 16 h of fasting, then began to accumulate substantial amounts of lipid after approximately 20 h. This accumulated lipid pool was selectively enriched in long-chain polyunsaturated fatty acids (LCPUFAs). Importantly, the authors showed that the lipid accumulation was driven primarily by increased uptake and reesterification of circulating FAs rather than by reduced lipolysis within BAT, as evidenced by remarkably increased expression of esterification-associated genes and unaltered hormone-sensitive lipase activation. These observations revealed an unexpected aspect of fasting adaptation: rather than immediately burning all the incoming FAs, BAT directs a substantial fraction of them into storage.

These findings raise an intriguing question: why would a highly oxidative tissue such as BAT actively store FAs during a period of nutrient deprivation? The answer began to emerge from the authors’ investigation of bone morphogenic protein 4 (BMP4), a well-established regulator of adipocyte differentiation and lipid metabolism (6). BAT BMP4 expression closely tracked the fasting-induced pattern of lipid accumulation, increasing substantially after approximately 20 h of fasting. This temporal association suggested that BMP4 might participate in the lipid and metabolic remodeling of BAT during prolonged fasting. To test this possibility, the authors generated mice with BAT-specific deletion of *Bmp4*. Strikingly, loss of BMP4 enhanced mitochondrial biogenesis in BAT and systemic energy expenditure. These effects were accompanied by increased FA oxidation and were dependent, at least in part, on AMP-activated protein kinase (AMPK), a central regulator of cellular energy metabolism (7). These findings suggested that BMP4 may function as an important molecular brake on the transition from FA esterification toward oxidative metabolism during fasting (Fig. 1).

**Fig. 1:**
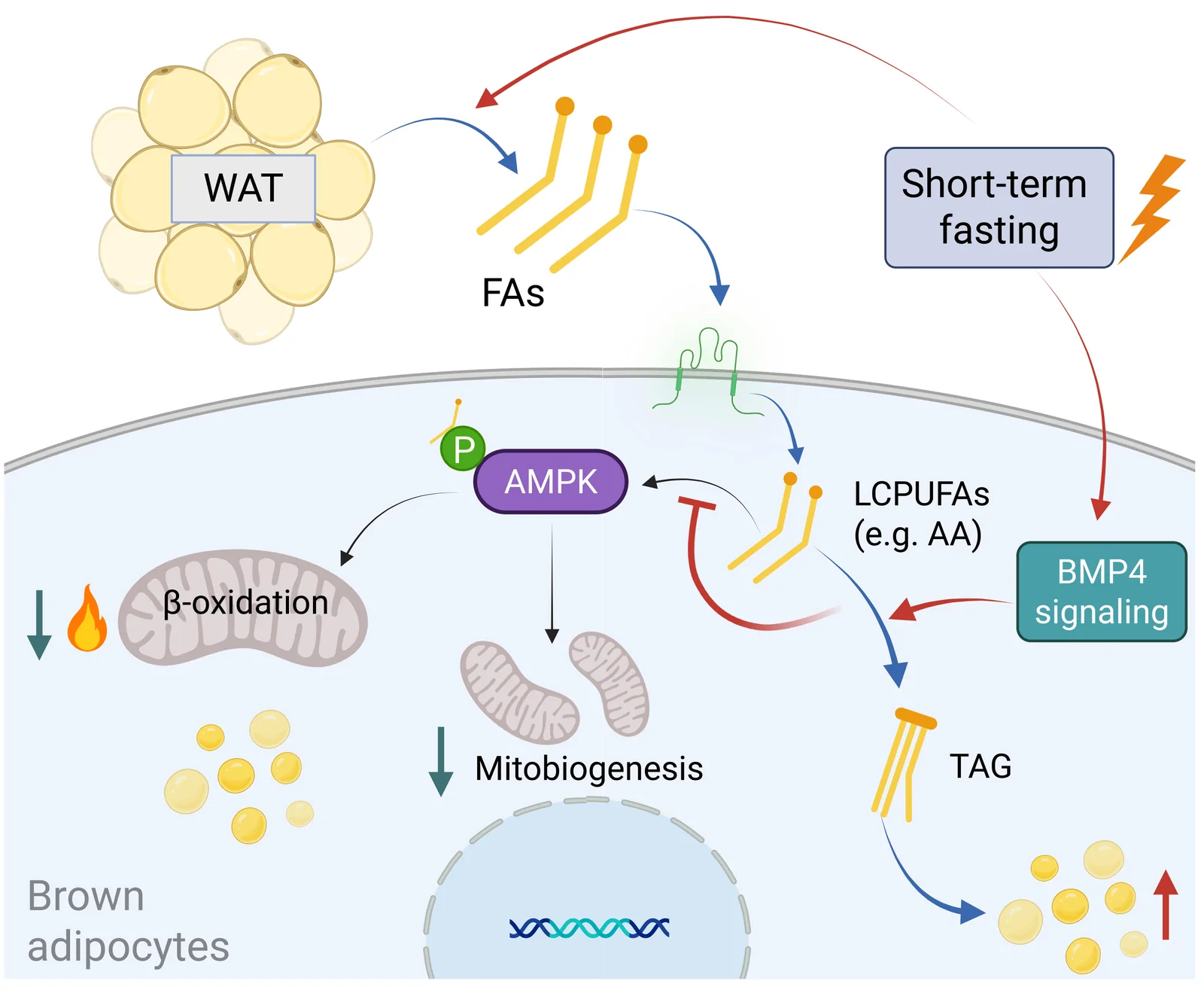
Short-term fasting triggers bone morphogenic protein 4 (BMP4)-mediated arachidonic acid (AA) reesterification in brown fat to limit excess FA oxidation and thermogenesis. Song *et al.* reported that during short-term fasting, brown adipocytes take up circulating free FAs released from white adipose tissue (WAT), with this lipid pool becoming enriched in long-chain polyunsaturated fatty acids (LCPUFAs). In parallel, BMP4 is induced to promote FA esterification, converting these released free FAs into triacylglycerol (TAG) for temporary storage in brown fat. Using a brown adipocyte–specific *Bmp4* knockout mouse model, the authors demonstrated that BMP4-driven FA esterification is not simply a mechanism for limiting the availability of substrates for mitochondrial oxidation and energy expenditure. Rather, it actively decreases the availability of free AA, an LCPUFA that, in its free form, can function as a signaling lipid that directly binds to and activates AMP-activated protein kinase (AMPK) by stabilizing its active conformation. Through this mechanism, BMP4-mediated FA esterification suppresses AMPK signaling, thereby limiting mitochondrial biogenesis and FA β-oxidation in brown adipocytes and preventing excessive adaptive thermogenesis.

The next question then became: what factors link altered FA storage to mitochondrial adaptation and BAT activation? To identify candidate signals, the authors integrated lipidomic analyses of circulating FAs, FAs esterified in BAT, and FAs whose esterification was reduced in *Bmp4*-deficient BAT during fasting. This approach narrowed the candidates to a particularly interesting lipid: arachidonic acid (AA), an LCPUFA that was consistently represented across these lipid pools. The authors demonstrated that loss of BMP4 increases the availability of free AA in BAT by suppressing its reesterification. Subsequent mechanistic studies revealed that AA binds to AMPK to stabilize its active conformation, thereby promoting mitochondrial biogenesis and FA oxidation to fuel thermogenesis (Fig. 1). These observations suggest that FA reesterification may serve as a mechanism to restrain availability of free FAs such as AA, thereby limiting their ability to activate oxidative metabolism in BAT. In this context, AA emerges not simply as a metabolic substrate but as a signaling lipid that directly promotes AMPK activation to facilitate mitochondrial biogenesis and FA oxidation. These findings also provide another example of how specific FAs can allosterically activate AMPK, expanding beyond the previously described role of long-chain fatty acyl-CoA esters and linoleic acid (8, 9).

Collectively, this study establishes a previously unappreciated link between lipid storage and thermogenic adaptation in BAT. Rather than viewing esterification and oxidation simply as competing endpoints of FA metabolism, the study suggests that the two processes can be functionally coupled. During fasting, BAT initially captures and reesterifies circulating FAs, creating a lipid reservoir enriched in signaling-competent LCPUFAs such as AA. Suppression of this storage pathway increases free AA availability, which activates AMPK and promotes mitochondrial biogenesis and thermogenesis (Fig. 1). In this model, transient lipid storage in BAT during fasting is not simply a consequence of metabolic demand. Rather, transient lipid storage in BAT serves as an active signaling mechanism that shapes subsequent oxidative metabolism independently of the conventional balance between lipogenesis and lipolysis. Moreover, this work identifies BMP4 as an important regulator of this metabolic switch. Previous studies have established BMP4 as a critical regulator of adipocyte development and metabolism (6), but its role in coordinating lipid flux and mitochondrial adaptation in mature BAT during fasting was less clear. The findings by Song *et al.* expand the functional repertoire of BMP4 and place it at the intersection of lipid esterification, FA signaling, and thermogenic adaptation.

These findings open several important areas for further investigation. First, why does fasting-induced lipid remodeling preferentially enrich AA and other LCPUFAs in BAT? The mechanisms that govern the selective uptake, esterification, and release of individual FA species remain elusive. It will be particularly interesting to determine whether AA functions as a dedicated fasting signal or whether other lipid species can engage similar AMPK-dependent pathways under different physiological conditions. More broadly, these findings raise the possibility that the identity of stored lipid species, rather than simply the total amount of lipid accumulated, may determine the downstream metabolic response.

Second, the mode of action of BMP4 in this pathway remain to be clarified. BMP4 is a secreted factor that typically acts through autocrine and paracrine mechanisms. Previous studies have shown that BMP4 is expressed at higher levels in the stromal vascular fraction than in mature adipocytes within BAT (10). Thus, deleting *Bmp4* specifically in mature brown adipocytes may not fully disrupt BMP4 signaling throughout the BAT depot. The enhanced AMPK activation, mitochondrial oxidation, and thermogenic capacity observed in the BAT BMP4-deficient mouse model therefore raise an important question: does BMP4 act primarily within mature brown adipocytes to regulate intracellular lipid metabolism, or does altered BMP4 within brown adipocytes indirectly influence the broader cellular environment of BAT? Defining the site of action and downstream signaling mechanisms of BMP4 will be important.

Third, the broader physiological relevance of this pathway remains to be established. Since both circulating and adipose-derived BMP4 have been implicated in the regulation of insulin sensitivity (11, 12), an important next step will be to determine whether BMP4 derived from brown adipocytes also contributes to glucose homeostasis during fasting. Dissecting how this pathway coordinates lipid and glucose metabolism could provide a more comprehensive understanding of BAT metabolic reprogramming during nutrient deprivation.

Finally, beyond fasting, BAT also undergoes substantial changes in lipid availability and mitochondrial demand in response to exercise, caloric restriction, and obesity. Whether BMP4-dependent lipid remodeling and AA–AMPK signaling operate under these conditions will be important to determine. Establishing whether this pathway is conserved in human brown or beige adipose tissue will be critical for assessing its translational relevance. Addressing these questions could reveal whether the mechanism described by Song *et al.* represents a fasting-specific adaptation or a broader strategy by which brown fat senses and responds to changes in lipid availability, potentially uncovering new therapeutic opportunities for metabolic diseases including obesity, hyperlipidemia, and type 2 diabetes.

## Conflicts of interest

The author declares that they have no conflicts of interest with the contents of this article.
